# RNA-seq in grain unveils fate of neo- and paleopolyploidization events in bread wheat (*Triticum aestivum *L.)

**DOI:** 10.1186/gb-2011-12-12-r119

**Published:** 2011-12-02

**Authors:** Caroline Pont, Florent Murat, Carole Confolent, Sandrine Balzergue, Jérôme Salse

**Affiliations:** 1 INRA, UMR 1095, Genetics, Diversity and Ecophysiology of Cereals, 234 avenue du Brézet, 63100 Clermont-Ferrand, France; 2 INRA, Unité de Recherches en Génomique Végétale, 2 rue Gaston Crémieux, CP 5708, F-91057 Evry Cedex, France

## Abstract

**Background:**

Whole genome duplication is a common evolutionary event in plants. Bread wheat (*Triticum aestivum *L.) is a good model to investigate the impact of paleo- and neoduplications on the organization and function of modern plant genomes.

**Results:**

We performed an RNA sequencing-based inference of the grain filling gene network in bread wheat and identified a set of 37,695 non-redundant sequence clusters, which is an unprecedented resolution corresponding to an estimated half of the wheat genome unigene repertoire. Using the *Brachypodium distachyon *genome as a reference for the Triticeae, we classified gene clusters into orthologous, paralogous, and homoeologous relationships. Based on this wheat gene evolutionary classification, older duplicated copies (dating back 50 to 70 million years) exhibit more than 80% gene loss and expression divergence while recent duplicates (dating back 1.5 to 3 million years) show only 54% gene loss and 36 to 49% expression divergence.

**Conclusions:**

We suggest that structural shuffling due to duplicated gene loss is a rapid process, whereas functional shuffling due to neo- and/or subfunctionalization of duplicates is a longer process, and that both shuffling mechanisms drive functional redundancy erosion. We conclude that, as a result of these mechanisms, half the gene duplicates in plants are structurally and functionally altered within 10 million years of evolution, and the diploidization process is completed after 45 to 50 million years following polyploidization.

## Background

More than 40 years ago, based on a few protein sequences from vertebrates, Susumu Ohno proposed polyploidization as a major source of new biological pathways created from duplicated gene copies [1]. The vertebrate genomes can be considered as paleopolyploids that had become modern diploids by means of ancestral chromosome fusions as well as sequence divergence between duplicated chromosomes. Recent paleogenomic analyses in plants have confirmed and refined Ohno's conclusions and led to the identification of polyploid common ancestors, showing that present-day species have been shaped through several rounds of whole genome duplications (WGDs), small scale duplications (SSDs) as well as copy number variations (CNVs) of tandem duplicated genes followed by numerous chromosome fusion (CF) events leading to the their present-day chromosome numbers [2-4]. Duplicate genes that persisted in multiple copies diverged by differentiation of sequence and/or function. Overall, recurrent gene or genome duplications generate functional redundancy followed either by pseudogenization (that is, unexpressed or functionless paralogs), concerted evolution (that is, maintained function of paralogs), subfunctionalization (that is, partitioned function of paralogs), or neofunctionalization (that is, novel function of paralogs) during the course of genome evolution. Functional divergence either by subfunctionalization or neofunctionalization of duplicated genes has been proposed as one of the most important sources of evolutionary innovation in living organisms [5]. As a consequence, polyploidy followed by diploidization is a major mechanism that has shaped complex regulatory networks during the evolution of the plant genomes. However, the real impact of genome duplication on gene network evolution, by comparing ancestral pre-WDG networks to modern post-WGD networks, is not clear. Recent access to numerous sequenced plant genomes [4] now offers the opportunity to study, at an unprecedented resolution, the impact of WGD on gene and genome organization as well as regulation.

**Figure 1:**
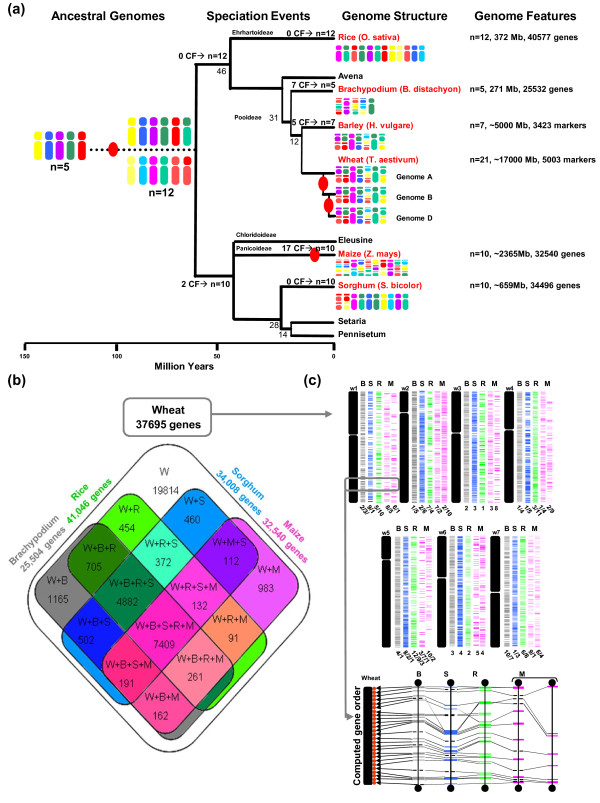
**Homolog gene conservation between wheat and cereal sequenced genomes**. **(a) **Cereal genome paleohistory. Schematic representation of the phylogenetic relationships between grass species adapted from [2,4]. Divergence times from a common ancestor are indicated below the branches of the phylogenetic tree (in million years). Whole genome duplication events are illustrated with red circles on the tree branches. The evolution of chromosome numbers of modern species from the ancestral genome structure is indicated with the number of chromosome fusion (CF) events. Genome features (number of chromosomes, physical size, and the number of annotated unigenes) of the six cereal genomes investigated are shown at the right-hand side. Modern genome architectures are illustrated using a color code that represents the *n *= 5 and 12 extinct ancestors (left). **(b) **Homologous gene groups between wheat and rice, *Brachypodium*, sorghum, and maize genomes. The Venn diagram illustrates the number of conserved protein domain-based homologs between wheat (RNA-seq gene clusters) and rice/*Brachypodium*/sorghum/maize (annotated proteins). **(c) **Simulated synteny-based gene order model in bread wheat. The chromosomal location of the RNA-seq gene clusters are shown on the seven bread wheat chromosome groups based on a consensus gene order derived from the observed synteny between wheat and rice ('R', in green), *Brachypodium *('B', in grey), sorghum ('S', in blue), and maize ('M', in pink) chromosomes (numbers are shown at the bottom of the chromosomes). The bottom inset illustrates a micro-synteny example of 26 re-ordered genes in bread wheat chromosome 1 (red dots) based on orthologous genes identified in *Brachypodium *(chromosome 2, 92 annotated genes, 0.9 Mb), sorghum (chromosome 9, 108 annotated genes, 1.1 Mb), rice (chromosome 5, 112 annotated genes, 0.9 Mb), maize (chromosomes 6 to 8, 145 annotated genes, 12.6 Mb). Non-conserved genes are illustrated using dotted lines and conserved genes are linked with black lines.

Recent paleogenomics studies in plants aiming at comparing modern genome sequences to reconstruct their common founder ancestors based on the characterization of shared duplication events allowed the characterization of seven genome paleoduplications for the monocots and seven genome paleotriplications for the eudicots. These data led to the construction of extinct ancestors of seven protochromosomes (9,731 protogenes) and five protochromosomes (9,138 protogenes) for the eudicots and monocots, respectively [4] (Figure 1a). These recent evolutionary studies in plants suggest that most duplicated genes that are structurally retained during evolution (referred to as 'persistent duplicated genes') have at least partially diverged in their function [6,7]. Microarray studies in eudicots and monocots showed that the vast majority of duplicated genes have diverged in their expression profiles, with 73% [8,9] and 88% [10] of gene pairs in *Arabidopsis *(eudicot reference genome) and rice (monocot reference genome), respectively, associated with asymmetric expression profiles after 50 to 100 million years of evolution. In maize, where a recent WGD dating back to 5 million years ago (MYA) occurred [11], more than 50% of the duplicated genes have been deleted and are no longer detectable within paralogous chromosomal blocks [12]. These results clearly demonstrate that most of the genetic redundancy originating from polyploidy events is erased by a massive loss of duplicated genes by pseudogenization in one of the duplicated segments soon after the polyploidization event.

Because many genes are part of more global regulatory networks, a change in the expression pattern of a single gene could induce changes for numerous genes involved in the same functional pathway. Haberer *et al. *[13] noted for example that tandem as well as segmental duplicate gene pairs exhibiting high *cis*-element similarities within promoters had divergent expression in *Arabidopsis*, suggesting that changes to a small fraction of *cis*-elements could be sufficient for neo- or subfunctionalization. We can argue that functional novelties derived from neo- or subfunctionalization of orthologous and paralogous copies may reduce the risk of extinction of plant species [14,15], similar to what has been suggested in mammals, where extinction events of vertebrate lineages is higher prior to the known ancestral WGD [16]. In this scenario, rapid genomic (that is, reciprocal gene loss) and functional changes (that is, neo- or subfunctionalization) following WGD might enable polyploids to better or quickly adapt to environmental conditions with improved physiological and morphological traits and properties that were not present or sufficient in their diploid progenitors. For instance, it has been suggested that neo- or paleopolyploidy may increase vigor [17], favor tolerance to environmental changes [15], and facilitate propagation through increased self-fertilization species [18,19].

To gain insight into the impact of genome doubling on gene structure and expression, we performed high-throughput RNA sequencing (RNA-seq)-based inference of the grain filling gene network in bread wheat. We focused our functional experiments on a grain developmental kinetic to be able to run comparable experiments in other cereals (for example, rice in the next sections) based on the main conserved grain developmental phases: cell division, filling, and dehydration. Bread wheat is a good plant model to study the impact of distinct rounds of WGD on gene structure and function, as its genome comprises seven ancestral paleoduplications shared with all known cereal genomes and two recent neopolyploidization events to form *Triticum aestivum*, which originated from two hybridizations, one between *Triticum urartu *(A genome) and an *Aegilops speltoides*-related species (B genome) 1.5 to 3 MYA, forming *Triticum turgidum *ssp. *durum*, and one between *T. turgidum *(genomes A-B) and *Aegilops tauschii *(D genome) 10,000 years ago [20,21]. Bread wheat is thus a good genome model to study in the same analysis the impact of ancient and recent WGD on genome structure and function. The bread wheat genome architecture offers us the opportunity to study not only the structures and corresponding expression patterns of paleoduplicated genes (50 to 70 million years of evolution) but also neoduplicated genes (1.5 to 3 million years of evolution) by comparing expression profiles of A, B and D homoeologous gene copies, that is, homoeoalleles (Figure 1a). As the complete assembled wheat genome sequence is not yet available, we have used *Brachypodium *as reference genomes to investigate the grain filling gene network modification in response to recent and ancient evolutionary events, such as duplication, polyploidization and speciation. The aim of this study was not to perform a quantitative (that is, transcriptome) analysis of the genes expressed during grain development but rather a robust qualitative identification (that is, large scale repertoire) of homoeologous/orthologous/paralogous gene networks, allowing us to provide new insights into the structural and functional evolution of genes after a WGD event in plants. This article provides relevant conclusions on how recent and ancient duplicated genes in plants evolve in both structure and function at the whole genome level, the gene family level, and the gene network level. The established divergence of structural and expression patterns between duplicated genes might have accelerated the erosion of colinearity between plant genomes as discussed in the article.

## Results

### Synteny-based gene repertoire and expression map in wheat

We performed an RNA-seq analysis of samples collected during the grain development in wheat. We used a 454 (Roche, see Materials and methods)sequencing platform with five developmental stages, that is, 100 degree days (DD), 200 DD, 250 DD, 300 DD, and 500 DD after pollination. The five developmental stages cover the cell division (100, 200, 250 DD) and filling (300, 500 DD) phases of grain development in wheat. RNA was extracted, pooled, and sequenced and sequence reads (934,928 in total) were clustered and checked for quality as described in the Materials and methods section in order to provide a qualitative and exhaustive view of the grain development gene network in bread wheat (Table S1 in Additional file 1). We obtained 37,695 sequence clusters (20.1 Mb of assembled sequences with an average coverage of approximately 25× per cluster) based on the assembly strategy protocol described in the Materials and methods section. Detailed information on the 37,695 sequence clusters (identity, sequence, and function) is available in Table S2 in Additional file 1 and consists of the most complete gene network repertoire of the grain development in wheat and probably in grasses more generally.

We aligned the 37,695 sequence clusters to the proteomes of the four monocot sequenced genomes, that is, rice, sorghum, *Brachypodium*, and maize (Figure 1b). Homologous gene pairs based on protein sequence conservation (BLASTx) of functional domains allowed us to establish that 17,881 (47%) wheat genes can be paired with a single homolog counterpart (based on sequence comparisons using 50% protein identity as a threshold criterion) in at least one of the considered sequenced genomes. The remaining 19,814 are putative wheat-specific unigenes (that is, not found in any of the four sequenced cereal genomes available to date) based on our BLAST alignment criteria, including 8,428 (43%) associated with wheat public EST-unigenes and 11,386 short reads (that is, an average of 430 bases for wheat-specific versus 650 bases for non-wheat-specific clusters) and/or low expressed/covered genes (that is, an average of 15× for wheat-specific versus 36× for non-wheat-specific clusers). We cannot finally exclude that such orphan clusters may correspond to sequenced poly-adenylated non nuclear sequences. As expected, the *Brachypodium *sequence genome appears to be the closest relative with the highest number of specific (not shared with any of the three other sequenced cereal genomes) protein-based homologs (1,165) identified in comparison with the wheat unigene set. A four genome-based synteny approach was used for all seven wheat chromosome groups by integrating wheat cytogenetic map information [22] and public chromosome-to-chromosome relationships [2,4] to produce the most parsimonious simulated gene order in wheat based on gene conservation observed among the four sequenced cereal genomes as detailed in Murat *et al. *[23]. Based on the known synteny relationship established between the seven wheat chromosome groups and the rice, sorghum, *Brachypodium *and maize genomes [23], we produced a partial wheat gene-based physical map where RNA-seq clusters were ordered within wheat chromosomes in respect to the position of their orthologous counterparts (following the ordering priority of rice >*Brachypodium *> sorghum > maize; Figure 1b; Table S3 in Additional file 1). A comparable approach has also been used recently in barley [24]. The gene content for chromosome 3B has recently been estimated to include 8,400 unigenes [25], of which 3,478 (41.4%) were available from the current analysis. We provide here the largest set of unigenes in wheat, covering almost half of the total genome-wide gene set based on the previous 3B chromosome comparison. Our wheat unigene set originated from a single tissue (grain), suggesting that only a few additional complementary ones (such as from root and leaf) would be sufficient to recover the vast majority of all genes in wheat. Therefore, we were able to place 17,881 wheat genes in a so-called computed or simulated order along chromosomes (Figure 1b) and have made the data available to users (Table S3 in Additional file 1) for further marker development or candidate gene identification. Figure 1c (bottom inset) illustrates the strategy used to infer computed gene order in wheat (chromosome group 1) based on the consensus gene order derived from the synteny observed between *Brachypodium *(chromosome 2), rice (chromosome 5), sorghum (chromosome 9) and maize (chromosomes 6 to 8) genomes. We therefore provide here for the first time the most complete qualitative set of unigene sequences expressed during the grain development in wheat associated with synteny-based physical locations on the seven chromosome groups.

Using the *Brachypodium *genome (5 chromosomes, 271 Mb, 25,504 gene models) as a reference to produce a heterologous wheat expression map, we could identify one-to-one robust orthologous gene pairs between wheat RNA-seq clusters and *Brachypodium *gene models using two nucleic acid alignment (BLASTn) parameters as described in Salse *et al. *[2,3] and the Materials and methods section. Briefly, with the BLASTn alignment based on default parameter (such as expect values), homologous gene relationships are obtained, although the analysis is polluted with background noise corresponding to high functional domain conservation, making it difficult to characterize which are the real significant single orthologous relationships between two considered genomes. The used parameters (CIP = 60% and CALP = 70%) return statistically significant single copy collinear relationships between two gene sets, and the remaining homologous gene relationships are then considered artifactual, that is, obtained at random [2]. Among the 37,695 RNA-seq clusters, 8,485 (23%, with an average size of 761 bases) wheat sequences could be aligned with 7,158 known orthologous genes in *Brachypodium *(Table S4 in Additional file 1) following this strategy. Map positions in wheat were simulated from syntenic relationships with *Brachypodium *as explained in the previous section. The remaining 29,210 RNA-seq clusters that could not be paired with *Brachypodium *gene models corresponded to short reads (average size of 468 bases) that were either considered as singletons or rejected based on our stringent sequence alignment criteria. These stringent alignment criteria were set to establish a robust repertoire of homoeologous/paralogous (wheat), orthologous (wheat/*Brachypodium*) genes in order to infer the consequence of evolutionary events (duplication, speciation) on gene structure and expression patterns, as discussed in the next sections. The objective of the current analysis was not to obtain the largest set of wheat homologous counterparts in *Brachypodium *for the 37,695 wheat sequence clusters (as described in the previous section and illustrated in Figure 1b) but rather precise and robust evolutionary relationships (conserved and duplicated genes) to investigate structural and functional redundancy.

In summary, we produced 37,695 wheat gene clusters (estimated to represent half of the total diploid wheat gene content based on the wheat chromosome 3B-based inference), of which 47% were associated with functional domain-based homologs of the sequenced genome proteomes (*Brachypodium*, rice, sorghum and maize) and 23% were strict orthologs with *Brachypodium*, considered as the sequenced reference genome for the Triticeae.

### Evolutionary fate of duplicated genes at the whole genome level

**Figure 2:**
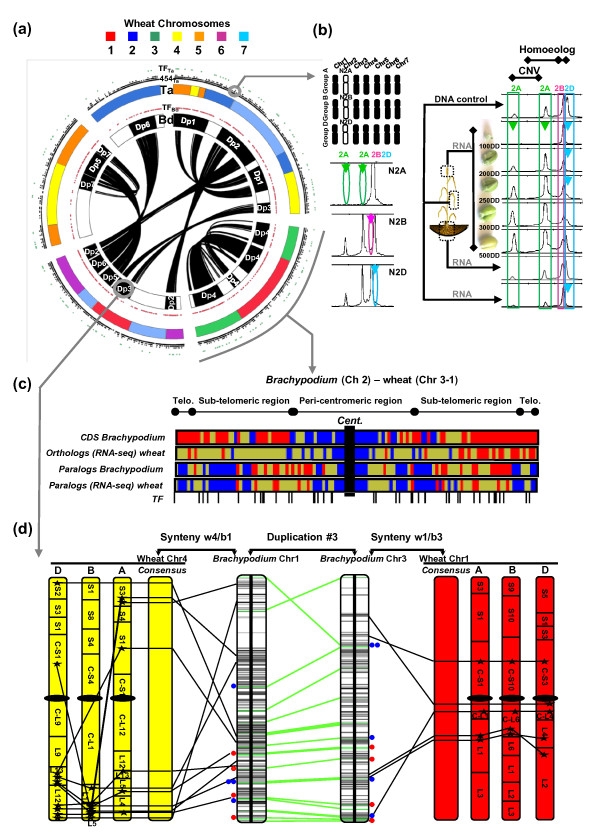
**Heterologous genome-wide wheat expression map**. **(a) **The five *Brachypodium *chromosomes are illustrated in the inner circle (labeled Bd) and the seven paleoduplications (in black) shared within cereals are displayed in the center (Dp1 to Dp7). The second circle (labeled Ta) illustrates the orthologous relationships identified between *Brachypodium *and wheat using a seven color code. Dots around the two circles illustrate the *Brachypodium *transcription factors (red; labeled TF_Ta_), the 454 RNA-seq reads from bread wheat (black; labeled 454_Ta_) and associated TFs (green; labeled TF_Bb_). **(b) **CNVs and homoeologous gene localization in bread wheat is illustrated with a single COS marker (CT753726) that has been located on chromosome 2A (CNV of 2), 2B and 2D using the adapted cytogenetic material illustrated in the top. The arrows illustrate the observed amplification loss observed for the illustrated cytogenetic material (N2A, N2B, N2D respectively for the absence of the 2A, 2B, 2D chromosomes). The COS marker (CT753726) expression (SSCP amplification) profiles observed in the five RNA samples from wheat grain development, as well as in leaves (RNA and DNA amplifications) and roots (RNA) considered as negative control. Colored arrows highlight the loss of expression of the considered CNV or homoeologous copies. **(c) **Wheat (chromsomes 1 to 3) and *Brachypodium *(chromosome 2) heat maps for *Brachydopium *coding sequence (CDS; blue < 40, yellow approximately 41 to 50, red > 51 genes/500 kb), wheat RNA-seq ortholog (blue < 9, yellow approximately 10 to 19, red > 20 genes/500 kb), *Brachypodium *paralog (blue = 0, yellow approximately 1 to 5, red > 6 genes/500 kb), wheat RNA-seq paralog (blue = 0, yellow approximately 1 to 3, red > 4 genes/500 kb) distributions. The 44 RNA-seq TFs are illustrated with their corresponding orthologous positions on the *Brachypodium *chromosome as black vertical bars. **(d) **Paralogous chromosomal regions are shown in the center, involving *Brachypodium *chromosome 1 (2.1 Mb, 252 genes) and 3 (2.9 Mb, 181 genes), and annotated genes are shown with horizontal bars. Orthologous wheat chromosomes are shown at the right (consensus group 1 and homoeologous chromosomes in red) and left (consensus group 4 and homoeologous chromosomes in yellow). Orthologous genes identified between wheat and *Brachypodium *are linked with black lines. Paralogous genes identified between *Brachypodium *chromosomes are linked with green lines. Expression data from wheat RNA-seq cluster alignment against the considered *Brachypodium *chromosome sequences are shown with colored dots. Blue dots illustrate paralogous gene pairs for which only one copy is associated with wheat RNA-seq clusters while red dots illustrate paralogous pairs for which both duplicates are associated with wheat RNA-seq clusters.

We produced a heterologous wheat expression map where 8,485 genes that were expressed during wheat grain filling were mapped strictly to the *Brachypodium *genome and positioned within the wheat genome based on the recently established *Brachypodium */wheat genomes colinearity [4] (Figure 2a). This heterologous wheat expression map (Table S4 in Additional file 1) has been used to study and discuss the evolutionary fate of paralogous, homoeologous and orthologous gene copies. Figure 2a depicts the five *Brachypodium *chromosomes as the inner circle (labeled 'Bd') and illustrates the seven paleoduplications (in black) shared with other cereals in the center [2,3]. The second circle (labeled 'Ta') illustrates the orthologous relationships identified between *Brachypodium *and wheat using a seven color code [4], illuminating the Triticeae chromosome group origins. Black dots around the wheat circle illustrate the 454 RNA-seq reads from wheat (labeled '454_Ta'_).

Our data clearly show that 6,024, 941, and 193 gene models matched with 1, 2, and 3 homoeologous gene copies in wheat, respectively (Figure 2a, 454Ta circles). Overall, only 193 of 7,158 orthologous gene pairs identified between *Brachypodium *and wheat matched the three expected homoeologous counterparts in wheat. Therefore, we can suggest that 2.7% of the homoeologous copies derived from two rounds of polyploidization that took place less that 1.5 to 3 MYA [20,21] have been structurally and functionally conserved in wheat. We cannot exclude that the expression of some homoeologs may be too low to be detected by RNA-seq given the coverage used in the current analysis, but we have clearly established that, for 6,024 genes with detectable expression signals, the three homoeologs do not have perfectly redundant expression profiles in the considered grain experiment. This clearly suggests that, for a large majority of the homoeologs in wheat, at least one copy has been lost (deleted or pseudogenized) or neo- and/or subfunctionalized within 1.5 to 3 million years of evolution. Moreover, Figure 2a illustrates that the genes expressed during the grain development were randomly distributed on the *Brachypodium *genome. Because of the average RNA-seq cluster length, however, we cannot distinguish homoeologous copies that have SNPs (1 per approximately 500 base-pairs in wheat) outside of the aligned sequences, leading to an overestimated percentage of homoeologous gene rearrangement events through homoeologous gene assemblage in the same cluster. We can still hypothesize that such homoeologs that do not harbor homoeoSNPs within the sequenced regions have been clustered together, leading to increased sequence coverage of the considered clusters. Consequently, we can extrapolate the homoeologous representation within a sequence assembly cluster based on the sequence coverage. The assumption is that for a sequence fraction of a gene that does not harbor homoeoSNPs, the three homologs are then clustered within the same assembly, leading to an increase in the sequence coverage of such a region. Monitoring putative merged homoeologs in the same clusters based on the sequence coverage of the initial 37,695 RNA-seq unigenes, we were then able to identify 1,009 clusters covered with more than 140 reads (that is, putatively merged homoeologs) compared to 7,158 single copy homologs reported previously and associated with an average coverage of 38 to 42 reads. We confirm at the whole gene repertoire level (37,695 non-redundant sequence clusters), following a sequence coverage-based approach for detecting homoeologous copies, what we observed initially using a synteny-based approach (2.7% of wheat homoeologs are associated with a *Brachypodium *orthologous gene), that 2.7% (1,009) of the homoeologous copies derived from two rounds of polyploidization that took place less than 1.5 to 3 MYA have been structurally and functionally conserved.

We designed an experiment using a subset of wheat genes to confirm the *in silico *structural and functional inference of homoeologous gene copies based on an *in omic *complementary approach. It is possible that missing data (non-sequenced low expression genes) have led to an overestimation of the structural and expression differences between homoeoalleles. If we select a subset of 100 genes, we should observe, based on the *in silico *conclusions detailed in the previous sections, that a vast majority of the homoeologs do not share the same expression pattern during grain development. To do so, from a set of 100 wheat genes randomly distributed among the 7 chromosome groups, we were able to design 91 primer pairs for further *in omic *structural (that is, evidence of homoeolog deletion) and functional (that is, evidence of homoeolog neo- and subfunctionnalization) inference of homoeologous gene copies in bread wheat. The Single Strand Conformational Polymorphism (SSCP) detection allows identification of homoeologous amplicons in a polyploidy background through the exploitation, on a capillary sequencer, of secondary DNA structure under non-denaturing conditions. The SSCP approach on a capillary sequencer [26] offers two advantages, the ability to detect SNP and size polymorphisms and to identify homoeologous or even paralogous amplifications. Using the wheat cytogenetic material available for the structural detection of putative homoeologs based on the SSCP technique, we observed that 43 (54%) out of 79 successfully assigned genes exhibited loss of at least one homoeologous copy beyond technical detection. Regarding expression patterns, 33 genes (36%) out of 91 showed a loss of expression when considering grain development, whereas 45 (49%) showed a loss of expression at the whole tissue level when comparing the expression in grain, the leaf, and the root (Figure 2b; Table S5 in Additional file 1). Consequently, 49% of the wheat homoeologous gene copies have been neo- and/or subfunctionalized when considering the grain developmental kinetic. Finally, only 27 (34%) genes out of 79 homoeoalleles detected on the three chromosome groups clearly show a conserved expression pattern in grain. The remaining 66% have either been structurally lost and/or neo- and/or subfunctionalized in their expression profiles. Figure 2b (left) illustrates the chromosomal localization of a single COS (conserved orthologous set [26]) gene (wheat CT753726 with rice ortholog LOC-OS04g33150) assigned to chromosome group 2 (homoeologous copies A, B and D as well as a single CNV for the A homoeolog). The same COS gene used to amplify, through the SSCP approach, the five RNA samples clearly shows that either the homoeologs (A, B and D copies) or CNVs do not present a perfect redundancy in their expression patterns. Figure 2b (right) illustrates how homoeoalleles and CNV expression signals were alternatively lost during grain development (colored arrows). Therefore, if 66% of homoeologs in wheat were either structurally lost (54%) or have diverged in their expression patterns (36% within tissues and 49% between tissues), earlier *in silico *assessments of homoeologous gene shuffling (that is, only 2.4% of homoeoalleles show conserved expression profiles) deduced from the alignment-based construction of homoeologous RNA-seq clusters was indeed overestimated (by about 20 to 30%), probably because of an average sequence read length of 761 bases as well as the possibility of missing low expressed genes, limits associated with this sequencing strategy.

Figure 1a illustrates a non-random distribution of wheat/*Brachypodium *orthologous genes at the whole genome level. As an example, Figure 2c shows *Brachypodium *chromosome 2, where the first heat map (coding sequence ('CDS') track) illustrates the distribution of annotated CDS with a clear enrichment of CDS in sub-telomeric regions (that is, 107.2 genes/Mb) and a reduced density in peri-centromeric regions (that is, 65.3 genes/Mb) due to transposable element (TE) invasion [27]. The second heat map illustrates the density of *Brachypodium *genes associated with a wheat ortholog ('Orthologs' track) based on the data set of 8,485 RNA-seq clusters (Table S6 in Additional file 1). The gene conservation is higher in peri-centromeric regions (31.1% of conserved genes) compared to telomeric (23.8% of conserved genes) or sub-telomeric (28.1% of conserved genes) regions. Finally, the paralogs (either *Brachypodium *or wheat gene 'paralogs' tracks) are not randomly and homogeneously distributed among chromosomes, that is, 47.4% versus 79.2% of duplicated genes in telomeric versus sub-telomeric regions, respectively. The 862 duplicated genes in *Brachypodium*, which arose from the seven ancestral duplications shared by the Poaceae, are depicted in the center of Figure 2a (from 1 to 7). Therefore, 166 *Brachypodium *paralogous pairs (19.3%) matched with their duplicated counterpart in wheat. The remaining 696 paralogous pairs (80.7%) matched with no or only one wheat sequence derived from the RNA-seq repertoire. This result is consistent with previous results [10] showing that 87.4% of the paleoduplicated genes in rice have been lost within a 50 to 70 million years of evolution. Figure 2d provides a detailed view of the ancestral duplication referenced as 'Dp3' shared between *Brachypodium *chromosomes 1 to 3 and wheat chromosomes 1 to 4. Duplicated genes are connected with a green line at the center of Figure 2d and wheat RNA-seq clusters that are orthologs of *Brachypodium *duplicated genes are illustrated with blue dots (wheat homoeologous genes identified for only one of the *Brachypodium *duplicates) or red dots (wheat homoeologous genes identified for both of the *Brachypodium *duplicates). At a micro-scale level for one (Dp3) of the seven ancestral duplications, among 20 paralogous gene pairs (green lines), 4 (20%) matched wheat homoeologous gene copies expressed during grain development (Figure 2d, red dots). This result further refines the conclusion that at either the whole genome level (19.3% of duplicates with concerted expression in the grain) or the micro-scale level (20% of duplicates with concerted expression in the grain), most of the paleoduplicated genes have been either lost or neo- and/or subfunctionalized so that the expression patterns at the tissue level are no longer redundant.

In summary, despite limitations of the RNA-seq approach in detecting low expressed genes and differentiating homoeoalleles, we have clearly shown at the whole genome level, using a heterologous wheat expression map, that almost 70% of recent duplicates (from homoeologous copy evolutionary analysis) have diverged during 1.5 to 3 million years of evolution (54% of homoeologous copies structurally lost and 36 to 49% of homoeologous copies with different expression profiles), and that more than 80% of ancient duplicates (from paralogous evolutionary analysis) have diverged during 50 to 70 million years of evolution.

### Evolutionary fate of duplicated genes at the gene family level

Out of the 7,158 *Brachypodium *genes corresponding to 1, 2, or 3 wheat homoeologous gene copies derived from the grain RNA-seq data described previously, 5,967 (corresponding to 7,112 wheat sequences) follow a canonical Gene Ontology (GO) classification. Among the 38 GO categories (from 'molecular function' classification) described at the whole genome level in *Brachypodium*, the distribution of three classes were shown to be statistically (based on chi-square test using 1% as a threshold) biased between grain development data (that is, from wheat RNA-seq) and what is observed at the whole-genome level (that is, annotated genes in *Brachypodium*): protein binding, transcription factor activity, and electron carrier activity (Table S7 in Additional file 1). The three previous GO classes are then good candidates to study the evolutionary fate of duplicated genes at the gene family level.

We recently performed a transcriptome analysis of rice grain filling based on an oligonucleotide array, where among the 60,727 genes spotted on the array, 29,191 were expressed during grain development [10]. In particular, we conducted a detailed analysis of 32 transcription factors (TFs) that were expressed during rice grain development. Across 100 to 600 gene physical intervals covering the entire rice genome, no co-regulation was observed between the selected TFs and the flanking genes [10]. In order to test this hypothesis, we conducted a specific analysis of TF gene families in wheat. Among the 666 TFs identified in the *Brachypodium *genome annotation (Figure 2a, TF_Bb _red dots), 161 wheat homoeologs were extracted from the RNA-seq clusters (Figure 2A, TF_Ta _green dots). Of these 666 *Brachypodium *TFs, 140 (21%) matched with a wheat ortholog that was expressed during grain development. Figure 2c shows a classical heat map representation of *Brachypodium *chromosome 2, including the distribution of 44 TFs (Table S8 in Additional file 1) from the wheat RNA-seq clusters that matched an orthologous counterpart of *Brachypodium *chromosome 2 (highlighted with black bars). As can be observed, whereas the distribution of genes among *Brachypodium *chromosomes is concentrated in the subtelomeric regions (see the previous section), the TFs are conserved in orthologous positions along the entire chromosome (that is, 0.5 TF/Mb in telomeric regions versus 0.6 TF/Mb in centromeric regions). These data complement and refine earlier conclusions about wheat diploidization-resistant genes, that is, genes that are preferentially conserved among cereal after WGD are TFs or TF-related gene functions [3,28], leading to a random distribution of this gene family in modern genomes.

In summary, we have established that, at the gene family level and using the TF family as a reference, that the GO 'transcription factor activity' class could be considered a diploidization-resistant gene function as it might have provided a selective advantage during evolution and adaptation and then retained as functional after WGD. Our data support preferential structural conservation of duplicated genes involved in signal transduction and more precisely transcription, which are putatively involved in response to rapidly changing biotic and abiotic extrinsic factors compared with genes encoding products involved in relatively more stable processes.

### Evolutionary fate of duplicated genes at the gene network level

In order to compare the wheat grain filling gene network with the previously published rice transcriptome analysis [10] described in the previous section, we conducted a similar analysis using the wheat Affymetrix array (based on the design and methods described in Wan *et al. *[29]). To avoid any bias due to different expression analysis methods - that is, RNA-seq versus Array technologies - we used the same RNA samples from the five wheat grain stages for hybridization of the wheat Affymetrix array (based on two independent biological replicates), see Materials and methods. Among the 6,760 rice/wheat transcripts identified between the rice (60,726 oligonucleotide probes) and wheat (61,115 oligonucleotide probes) arrays, 2,600 (38.4%) showed concerted (that is, Presence versus Absence Variation, referenced as PAVs) expression signals during grain development (Table S9 in Additional file 1). When considering not only rice/wheat orthologs but also paralogs that might have conserved the original or ancestral gene function and expression in rice and wheat, the percentage of concerted expression between both species would increase to 43.5% (that is, 2,944 genes).

**Figure 3:**
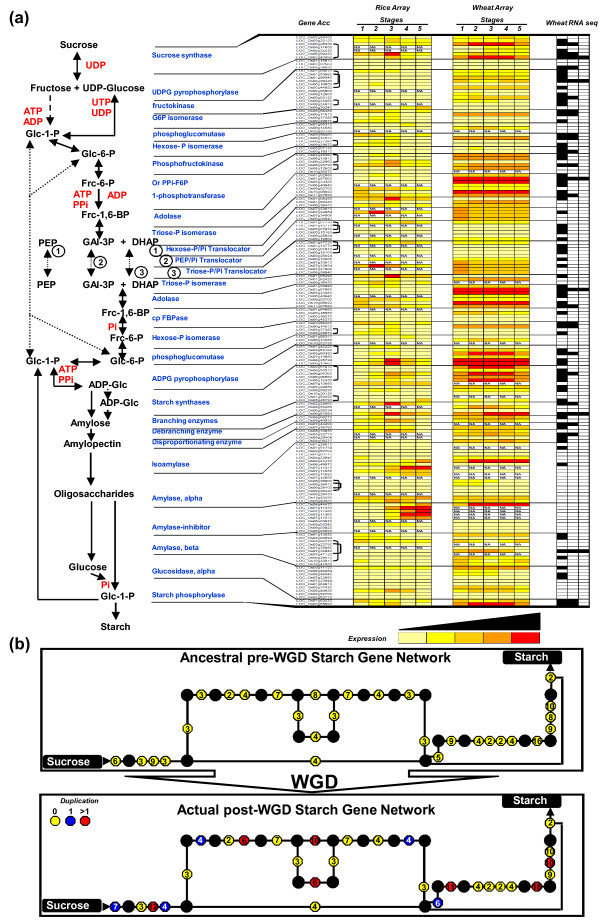
**Comparative evolution of the starch gene network between rice and wheat**. **(a) **Twenty-nine enzyme-encoding genes involved in the starch biosynthesis network are listed at the left and 170 genes (named according to their accession numbers) are shown. Expression data from oligo-array experiments performed in rice and wheat are displayed using a classical colored scale in which yellow illustrates the absence of expression and red the observed highest level of expression (6.5 for rice, 14.5 for wheat as normalized intensity). At the right are illustrated orthologous RNA-seq clusters from one up to three homoeologous copies (black scares). **(b) **The starch gene network is schematically represented with black dots representing synthesized substrate and the number of gene-encoding enzymes is shown in circles. The pre-WGD (top) and post-WGD (bottom) networks illustrate the impact of paleoduplication where no impact (yellow), gain of an additional enzyme-encoding gene (blue) and more than one additional enzyme-encoding gene (red) are shown.

Among the plant metabolic networks, the starch synthesis pathway is well known because starch is considered a major key regulator of grain development. In this network, 170 enzyme-coding genes can be represented with nodes and substrate-product metabolite flux by directional edges [30]. Figure 3 illustrates the comparative gene network observed between rice (gene profiles from microarray data [10]) and wheat (expression data from the current oligo-array and RNA-seq data) for the starch biosynthesis pathway described in Zhu *et al. *[31] (Table S10 in Additional file 1). Among the 170 genes involved in this network, 24 (14%) were identified as differentially expressed in rice and wheat based on the microarray experiments. However, based on the wheat RNA-seq data, 84 (49%) of the 170 enzyme-coding genes could be matched with 1 (57 genes), 2 (21 genes) or even 3 (6 genes) homoeologous copies. We also could show that among the 84 genes for which we have identified RNA-seq clusters as proof of expression in grain development, only 6 (7%) matched their three homoeologous counterparts. This micro-scale analysis, focused on a unique and specific well-known gene network, also agreed with the whole-genome level analysis that revealed that, for a large majority of the homoeologs in wheat, at least one copy had been lost or neo- and/or subfunctionalized during 1.5 to 3 million years of evolution.

Figure 3b illustrates the impact of the paleoduplication in grasses on the starch network. Based on the identification of 20 duplicated genes (black brackets in Figure 3a, 'Gene Acc' column) within the 170 enzyme-coding genes, we can suggest that 12% of the actual modern post-WGD network has been enriched by the ancestral shared tetraploidization event. We can then model the ancestral pre-WGD network consisting of 150 non-redundant starch enzyme-coding genes (Figure 3b). The observation that the post-WGD network is more abundant and enriched in TFs is also consistent with previously reported biases in gene functions after WGDs in plants [32,33] as well as in fungi and mammals [7,34]. Previous results for cereal genomes [2-4,28] and for eudicots [32] clearly showed that retained duplicated gene families correspond to transcriptional regulators that were preferentially conserved after WGD events. However, our analysis, based on a single gene network, did not confirm earlier reported conclusions in *Arabidopsis *that bottleneck enzymes in metabolic networks, which tend to connect different modules, are preferentially retained as functional duplicates after WGD [35]. In our case, of the seven genes preferentially retained as duplicated (highlighted in red in the post-publication network representation), none correspond to enzyme-node encoding genes.

In summary, we suggest that, at the gene network level and using the starch biosynthesis pathway as a reference, 14% of the rice-wheat orthologous copies have the same expression pattern (compared to up to 44% at the whole-genome level), 7% of the wheat homoeologous triplicates share the same expression pattern (consistent with what is observed at the whole-genome level), and WGDs have enriched the starch gene network by up to 12% in gene content.

### Evolutionary consequences of duplicates on genome colinearity

Structural rearrangement and gene loss between duplicated regions results in the reduction of orthologous relationships between cereal genomes. Duplicated gene loss in maize (Figure 1b, bottom inset) accounts for the major source of erosion of colinearity between maize and the other grass genomes. Gene colinearity observed between maize chromosome 8 (or 6) is reduced compared to the microsynteny observed between *Brachypodium*, rice, and sorghum at the same loci due to the recent WGD that occurred specifically during maize genome evolution. Only seven chromosome 8 (purple) and eight chromosome 6 (purple) genes are conserved between maize and the other three cereal genomes compared to the 26 orthologous relationships (grey, blue, green in Figure 1b) identified when comparing the rice, sorghum, and *Brachypodium *genomes.

**Figure 4:**
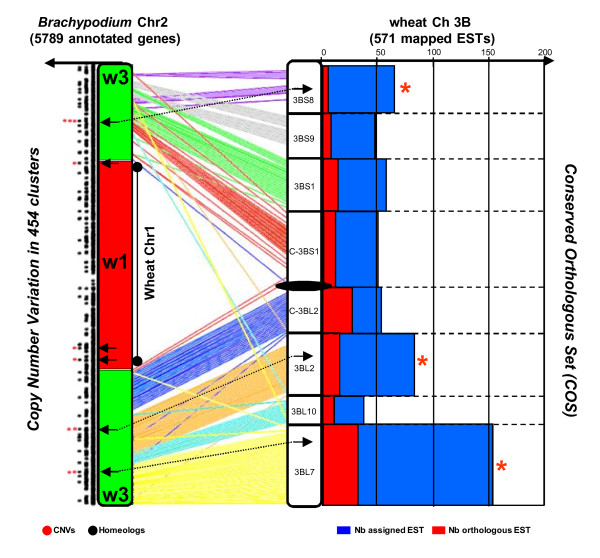
**Correlation between CNV and synteny erosion between *Brachypodium *and wheat**. At the right is illustrated the wheat chromosome 3B subdivided into eight deletion bins surrounding the centromere. The number of available mapped ESTs per bin is illustrated with blue bars whereas the number of conserved orthologous sets (COS) is shown with red bars. The three deletion bins associated with the lowest percentage of COS regarding the number of available mapped ESTs are indicated by red stars. At the left is illustrated the *Brachypodium *chromosome 2 subdivided into orthologous chromosomal relationships with wheat chromosomes 1 (in red) and 3 (in green). The wheat RNA-seq clusters are shown according to their orthologous positions on *Brachypodium *chromosome 2 as homoeologs (black dots) or additional CNVs (red dots). At the center are shown the *Brachypodium*/wheat orthologous genes linked with colored lines according to their position within the wheat deletion bins.

Despite the diploidization process following WGD associated with the loss of homoeolog and/or paralog sister gene copies being the major source of genome colinearity erosion, CNV is also an important phenomenon that can contribute to the observed reduced synteny between grass genomes, as illustrated in the Figure 1b where a single-copy COS gene identified in rice, *Brachypodium*, maize and sorghum corresponds to a putative CNV in bread wheat chromosome 2A. Such species-specific CNVs will not be associated with any orthologous counterpart in the other genomes, thus reducing the percentage of conserved and orthologous genes in grasses. Figure 4 illustrates the difference between the loss of synteny and the increased number of tandem duplications, which were referred to as CNVs. At the right-hand side of the figure, bread wheat chromosome 3B is shown with 8 deletion bins, for which the number of available ESTs (blue bars) as well as the number of orthologous genes (red bars) with *Brachypodium *chromosome 2 is illustrated. The orthologous blocks observed between wheat chromosome 3B and *Brachypodium *chromosome 2 are illustrated in different colors in the center of the figure. Finally, at the left-hand side of the figure, *Brachypodium *chromosome 2 is split into orthologous blocks of bread wheat chromosome 3B. The number of RNA-seq clusters of *Brachypodium *genes is depicted as circles (black for homoeologous copies and red for CNVs). A clear correlation between the loss of colinearity and increase of CNV can be observed. The three 3B bin intervals displaying the highest loss of colinearity (3BS8, 3BL2, 3BL7; indicated by red stars) are associated with orthologous regions of *Brachypodium *chromosome 2 comprising CNVs (linked with dotted black lines). Considering *Brachypodium *chromosome 2 as an example of a reference and model chromosomal structure, CNVs in wheat were preferentially located within subtelomeric regions of modern chromosomes or paleo-inserted chromosomes (that is, the ancestral fusion event between W3 in green and W1 in red). We suggest here that the loss of colinearity observed locally between *Brachypodium *and wheat is mainly due to tandem gene duplications putatively favored by recent polyploidization events in bread wheat.

In summary, we have shown that, at the whole genome as well as the chromosome level, segmental duplications and gene duplications in tandem (CNVs) comprise the main basis of colinearity loss between cereal genomes.

## Discussion

### Structural divergence between duplicated genes in plants

Our estimate of the frequency of chromosomal rearrangements (that is, duplicated gene loss) between homoeoalleles in wheat - 54% within less than 1.5 to 3 million years of evolution - needs to be viewed in the context of published studies. Qi *et al. *[22], based on a restriction fragment length polymorphism (RFLP) genotyping approach, mapped 7,104 EST unigenes onto 16,099 loci within the 21 bread wheat chromosomes. Because 39% of the ESTs mapped to the three homoeologous groups, those studies might have suggested that up to 61% of the homoeologs might have lost at least one of the homoeoalleles even despite technological limits due to the RFLP mapping resolution. Overall, we suggest here that, based on our and published data, 54 to 61% (depending on the genetic mapping or chromosome assignation procedures) of the wheat homoeoalleles have been entirely deleted or pseudogenized within less than 3 million years of evolution.

Re-analysis of the paleoduplication within the rice genome, consisting of ten major duplications as part of a WGD event 50 to 70 MYA, has shown that 87.4% of the duplicated genes have lost their orthologous counterparts [10]. Diverged polyploids, such as maize, are likely to have evolved from ancient polyploids by a process of pseudogene formation followed by sequence loss. In a study of the fate of duplicated genes in the maize genome, Lai *et al. *[36] and Messing *et al. *[12] have suggested that, within 5 million years of evolution, about 50% of duplicated genes have been lost through deletion. Nonetheless, gene duplication in maize, *per se*, via (auto)polyploidization may be associated with detectable increases in expression level, as demonstrated by Guo *et al. *[37]. Blanc *et al. *[9] reported similar findings from the *Arabidopsis *genome, where also only 20% of paralogs were retained within duplicated segments. More precisely, the authors stated that 28% and 13.5% of duplicated genes are retained in recent (date back to the *Arabidopsis*/Brassicacae divergence, 24 to 40 MYA) and old (date back to the monocot/dicot divergence, approximately 150 to 200 MYA) duplication blocks, respectively. Considering the recent data obtained in dicots (*Arabidopsis*) and monocots (rice, wheat, maize), our results provide additional support that most of the genetic redundancy originating from polyploidy is erased by a massive loss of duplicated genes by pseudogenization in one of the duplicated segments soon after the polyploidization event.

The structural loss of duplicated genes between paralogous segments as well as gene duplication in tandem (CNVs) accounted for a large part of the erosion of colinearity between cereal genomes. It became clear that using synteny-based approaches to establish a virtual gene order in non-sequenced genomes might mimic up to 77% of the gene order and content [4]. The remaining consists of lineage-specific duplicates loss and CNVs that will not be known until the genome is fully sequenced [4]. However, we can estimate that a large majority of the gene content can be modeled based on synteny, especially to support the development of gene-based markers such as COS [26].

### Expression divergence between duplicated genes in plants

As for chromosomal rearrangements, we also need to place our estimate of the frequency of change in expression patterns between homoeoalleles in wheat - 36 to 49% (depending on the considered tissues) within less than 1.5 to 3 million years of evolution - in the context of published studies. Using a similar cDNA-SSCP approach to that reported in this study, Bottley *et al. *[38] demonstrated that for 27% (in leaf) and 26% (in roots) of the considered genes, one homoeologous copy was not detectable within the cDNA samples. Our estimate of functional partitioning between homoeoalleles includes not only a presence/absence variation at the tissue level (49%) but also takes into account the difference in the expression profiles based on a developmental kinetic within a specific organ (36% in wheat grain). Using a cDNA-amplified fragment length polymorphism (AFLP) assay, Kashkush *et al. *[39] estimated that about 5% of the genes are silenced in a newly synthesized allohexaploid, a figure comparable with the study of He *et al. *[40] using a similar approach. This level is substantially lower than our estimates, but not surprising given the time gene silencing could continue over many generations. It certainly confirms that the diploidization process immediately follows the polyploidization event. Exploiting large collections of EST data, Mochida *et al. *[41,42] concluded that silencing affected 11 out of 90 sets of homoeoalleles (12%). Overall, based on our and published data, we suggest that 12 to 49% (depending on the tissues and approaches considered) of the wheat homoeoalleles have been neo- and/or subfunctionalized within less than 3 million years of evolution.

A similar difference between synthetic and ancient hybrids has been demonstrated in cotton, where Adams *et al. *[43,44] used a cDNA-AFLP assay to show that about 5% of all genes are silenced in a newly synthesized allotetraploid, but that about 25% of genes were affected in natural tetraploid cotton (within 1 to 2 million years of divergence). However, in newly synthesized *A. thaliana *× *A. arenosa *hybrids described by Comai *et al. *[45], only 0.4% of genes are silenced as a direct result of polyploidization, a figure substantially lower than that described in wheat and cotton. On the other hand, a survey on gene expression variation of *A. thaliana *and *A. arenosa*, which split from a common ancestor approximately 1.5 MYA, showed a higher number of approximately 2.5% of gene expression differences. The reason(s) for these disparities remain unclear, but might be a consequence of lower levels of homoeology between the two contributing genomes, and therefore the induction of a lower level of interference in their independent expression. Still, gene silencing certainly appears to be a common phenomenon in established polyploids, and the frequency of silencing seems to increase over time. Using detailed analysis of expression divergence between rice paleoduplicates, Throude *et al. *[10] have shown that 88%, 89%, and 96% diverged in their expression pattern in grain, leaf and root within 50 to 70 million years of evolution, respectively. Blanc *et al. *[9] showed that 57% (for young duplications) to 73% (for old duplications) of paralogs have diverged in expression based on a computational analysis involving 62 Affymetrix microarray experiments in *Arabidopsis*. However, Blanc *et al. *[9] cautioned that the 73% of gene pairs that have diverged in expression in the context of old duplications is an underestimate as cross-hybridization occurred at a high rate in this type of array-based experiment. Finally, expression of maize duplicates has been investigated through EST and cDNA mapping (EST-overgos by Gardiner *et al. *[46] and cDNA-RFLP by Helentjaris *et al. *[47]), suggesting that 20% and 29%, respectively, of the considered probes identified two distinct contigs or loci. These data suggest that 71 to 80% of the maize paralogs have diverged in their expression profiles from both EST and cDNA-based mapping experiments. However, gene silencing of duplicated copies rather than deletion is probably more a gene-dosage effect than just a strict diploidization response. Paralogous copies of prolamin genes (a medium size multigene family) in maize also showed that less than 50% of the duplicated copies remained intact [48]. Interestingly, at the same level, differential gene amplification (such as CNVs) also resulted in subfunctionalization of additional gene copies by divergent transcriptional regulation, mimicking the same events that happen in the same period of evolution between homoeologs [49].

### Temporal modeling of structural and expression gene shuffling after duplications in plants

**Figure 5:**
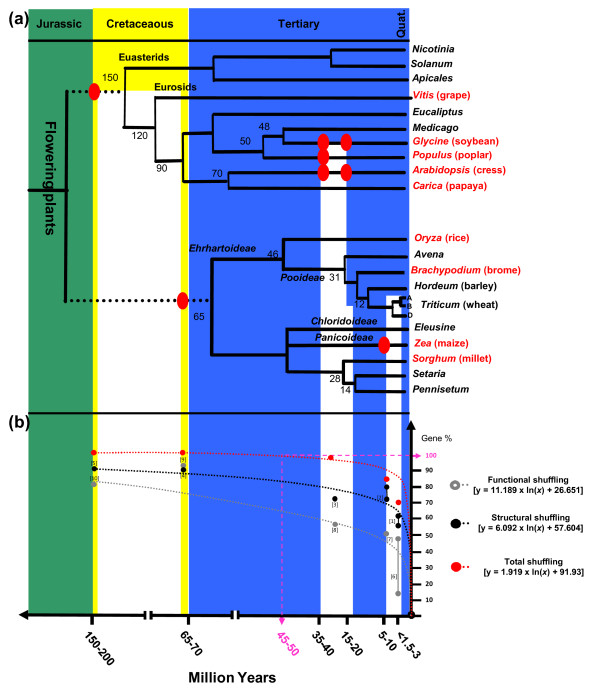
**Temporal model of structural and functional gene shuffling in plants**. **(a) **Schematic representation of the phylogenetic relationships between angiosperm species. Divergence times from a common ancestor are indicated on the branches of the phylogenetic tree (in million years). Sequenced genomes are indicated in red. WGD events are illustrated with red circles on the tree branches. **(b) **The percentage of total and structural (gene loss) or functional (neo-subfunctionalization) gene shuffling discussed in the article (referred to as data point 'X' in the text) and observed during the last 100 million years of evolution are plotted as dotted red, black and grey logarithmic curves.

We established clearly in this study that around 70% of homoeoalleles in the hexaploid wheat genomes have been lost (54 to 61%) or have diverged in gene expression (12 to 49%) since 1.5 to 3 MYA. These data confirm and complement the conclusion of Mochida *et al. *[41,42] that, considering 79 genes with scored expression in 10 tissues, 15 (19%) were expressed equally for the three homoeologs whereas the remaining 64 (81%) showed preferential homoeologous gene expression in at least one of the considered tissues. Based on the collective data from wheat detailed in the current article and from other plant species available in the literature, we tried to model the structural and functional consequences of gene set amplification after genome doubling for the last 100 million years of evolution. Figure 5a illustrates plant phylogeny, where speciation events are dated in MYA and known WGDs are marked with red dots. Based on our and other studies from the literature referenced in Table S11 in Additional file 1, we propose that structural rearrangements (from pseudogenization up to deletion) have been suggested to affect 54 to 61% of wheat homoeoalleles (Figure 5b, data point 1), 71 to 80% of maize neoparalogs (Figure 5b, data point 2), 72% of *Arabidopsis *neoparalogs (Figure 5b, data point 3), 87% of rice paleoparalogs (Figure 5b, data point 4), and 86% of *Arabidopsis *paleoparalogs (Figure 5b, data point 5) after 1.5 to 3, 5, 24 to 40, 70 to 100, and 150 to 200 million years of evolution, respectively. Regarding the impact of polyploidy on functional differentiation between duplicated gene copies, our and published data have suggested that 12 to 49% of wheat homoeoalleles (Figure 5b, data point 6), 50% of maize neoparalogs (Figure 5b, data point 7), 57% of *Arabidopsis *neoparalogs (Figure 5b, data point 8), 88% of rice paleoparalogs (Figure 5b, data point 9), and 73% of *Arabidopsis *paleoparalogs (Figure 5b, data point 10) do not exhibit any concerted and redundant expression after 1.5 to 3, 5, 24 to 40, 70 to 100, and 150 to 200 million years of evolution, respectively.

Given the prevalence of gene and genome duplication in the paleohistory of plant, species and lineage development in angiosperms might differ from organisms where genome duplication was rare and where extensive expression divergence following duplication would have a profound impact on the pattern of developmental and regulatory networks. Our data support the idea that after 50 to 70 million years of evolution since grass genomes experienced a shared paleotetraploidization event, the vast majority of the homoeologous genes have been lost within a sister block and that the expression profiles of the remaining gene copies have largely diverged (Figure 5b). Changes in gene expression may have occurred immediately after polyploidy or might need a few generations to reach a new expression status. This trend towards silencing (or gene loss via pseudogenization) or expression shift (via neo- or subfunctionalization) of a particular locus soon after a polyploid event could be advantageous for adaptation and the establishment of a successful polyploid genome compared to its diploid founder progenitor. Figure 5b shows the evolution of gene function (grey dotted curve) and structure (black dotted curve). It follows that loss of duplicated genes due to mutation and deletion appeared to be a rapid and exponential process arising immediately after polyploidy because there is sufficient time for point mutations to accumulate. Moreover, expression modification and silencing of duplicated genes appear to take longer and are probably epigenetically induced (that is, the putative causal factor). Strikingly, based on the deduced total duplicated gene shuffling inference (dotted curves in Figure 5b) within approximately 10 million years of evolution after a polyploidization event, approximately 50% of the homoeologs have either been lost or sub- or neofunctionalized in plants. The superimposition on these immediate or short-term (putatively mutation-based changes) and longer-term (putatively epigenetic-based changes) responses to genome doubling might explain the observed structural and functional partitioning among gene pairs originating from a duplication event. We can hypothesize that the diploidization that takes place immediately after a WGD is completed for 100% of the duplicated genes after 45 to 50 million years of evolution, the evolutionary timescale necessary to observe that none of the sister duplicated gene copies exhibit any structural or expressional or functional redundancy (pink dotted lines in Figure 5b).

Gene dosage relations, which play a huge role in genome reorganization, are unbalanced after a WGD due to function redundancy between duplicated copies. Structural and functional shuffling occurs relatively soon (within less than a few million years of evolution) after polyploidy in plants that are still cytogenetically polyploidy (such as in the case of bread wheat in the current study), but is still active several million years latter, during or after cytological diploidization (such as in the case of rice, *Arabidopsis *and maize in the current study). We may hypothesize that epigenetic differences between duplicates or even sub-genomes deriving from WGD might have contributed to a gene or genome dominance through the rapid differentiation of expression toward gene dosage balance recovery soon after polyploidy. Wang *et al. *[50] observed silencing of polyploidy-derived duplicates due to hypermethylation in *Arabidopsis *polyploids. Epigenetic mechanisms as well as interaction networks might be the origin of an extremely rapid divergence of expression between duplicated genes soon after polyploidization. It has even been reported that polyploidization-derived modulation of expression between gene pairs was due to epigenetic mechanisms (*sensu lato*) in higher plants (reviewed in [45,51]). Based on our data set and derived conclusions, the bread wheat genome could be considered as a pertinent model for studying the molecular basis of the interaction between homoeologous gene pairs, especially the epigenetic basis of such observed modification in expression between duplicates in response to polyploidizations. The spectrum of phenomena discussed here illustrates the immediate impact of polyploidy on genome structure and its profound implication for evolution. For example, some of the observed genomic changes are known to affect phenotypes in ways that are highly visible to natural selection. A case in point concerns genomics rearrangements that affect the flowering-time locus/network in synthetic *Brassica *polyploids. These polyploidy-induced structural and functional rearrangements may impact traits as relevant as flowering-time divergence in modern plant species.

## Conclusions

Even if our estimates of divergence of expression between gene pairs might represent an underestimation of the true values in wheat because the data set is (i) centered on a grain developmental kinetic and then only a sampling of possible environmental conditions or tissues where the duplicated genes may be expressed, and (ii) based on RNA-seq, which may bias low expressed gene and homoeoallele identification, one cannot escape the theme that a large majority of the polyploidy-derived duplicated genes in plants have acquired divergent expression patterns and with them probably functions. Overall, duplication-mediated structural and functional gene shuffling promote a powerful acceleration of evolution in plants.

## Materials and methods

### Plant material and RNA extraction

#### Plant material

Two hundred seeds of hexaploid wheat, *Triticum aestivum *(cv. *Récital*), were sown with 4/5 Neuhaus compost and 1/5 Pouzzolane. After 8 weeks of vernalization, plants were transferred to a greenhouse with normalized temperature (approximately 18.5°C), light and hygrometry conditions (60%). The main stem heads were tagged at anthesis and grain samples (endosperm and embryo) were collected at 100, 200, 250, 300 and 500 DD after pollination. Two biological replications of samples were done in 2004 and 2006. Leaves were sampled at different growing stages and pooled, and roots were sampled on 12-day-old seedlings grown in sand.

#### RNA extraction

Grain wheat (100, 200, 250, 300, 500 DD), root and leaf samples (approximately 1 g of tissue) were ground in liquid nitrogen and extracted with 4.5 ml of buffer (10 mM Tris-HCl, pH7.4, 1 mM EDTA, 0.1 M NaCI, 1% SDS) and 3 ml of phenol-chloroform-isoamyl alcohol mixture 25:24:1. The supernatant was extracted one more time with the same phenol solution in order to eliminate proteins and starch residues. The nucleic acids were precipitated by addition of 0.1 vol of 3M AcNA pH5.2 and 2 vol of 100% ethanol. After precipitation, RNA was rinsed once with 70% ethanol and the pellets dissolved in RNase-free water. Purification was made with a DNAse treatment RNase-Free DNase Set (Qiagen, [52]) and then an RNeasy MinElute Cleanup Kit (Qiagen). The integrity of RNA was checked with an Agilent 2100 Bioanalyser microfluidics-based platform using a RNA 6000 Nano Chip kit and reagents (Agilent Technologies, [53]).

### 454 sequencing and cluster assembly

#### Normalized cDNA library construction

mRNA was purified from 5 μg total RNA by exonuclease digestion followed by LiCl precipitation (mRNA-Only Eucaryotic mRNA Isolation Kit, Epicenter, [54]). mRNA (1 μg) was used for first-strand cDNA synthesis. cDNA synthesis and amplification were done according to the Mint-Universal cDNA Synthesis Kit user manual (Evrogen, [55]). Amplified cDNA (800 ng) was used as starting material in the normalization reaction using the Trimmer Kit (Evrogen), and normalized material was re-amplified for 18 cycles. Normalized cDNA (2 μg) was digested with 10 units SfiI for 2 hours at 48°C. Fragments larger than 800 bp were isolated from a LMP Agarose Gel and purified using the MinElute Gel Extraction Kit (Qiagen). Purified cDNA fragments (200 ng) were ligated to 100 ng using SfiI and dephosphorylated usin pDNR-lib Vector (Clontech, [56]) in 10 μl using a Fast Ligation Kit (NEB, [57]). Ligations were desalted by ethanol precipitation, and re-dissolved in 10 μl water. Three-fold 1.5-μl desalted ligation was used to transform NEB10b competent cells (NEB), and 96 clones were randomly selected for sequencing to verify successful normalization. Roughly a million clones were plated on LB-Cm plates, scraped off the plates and stored as glycerol stocks at -70°C. One half of the cells were used to inoculate a 300-ml Terrific Broth/Cm culture, which was grown for 5 hours at 30°C. Plasmid DNA was prepared using standard methods (Qiagen). Purified plasmid DNA (200 μg) was digested with 100 units SfiI for 2 hours at 48°C. cDNA inserts were gel-purified (LMP-Agarose/MinElute Gel Extraction Kit) and ligated to high-molecular-weight DNA using a proprietary SfiI linker.

#### Roche 454 FLX library preparation and sequencing of the cDNA concatenates

The five grain samples (100, 200, 250, 300, 500 DD) were equally mixed for sequencing library construction and sequencing with an approximately 30× gene coverage (based on 1 million reads per run and approximately 30,000 expressed genes obtained from the Affymetrix experiment on the same samples discussed in the Results section). Library generation for the 454 FLX sequencing was carried out according to the manufacturer's standard protocols (Roche, [58]). In short, the concatenated inserts were sheared randomly by nebulization to fragments ranging in size from 400 to 900 bp. These fragments were end-polished and the 454 A and B adaptors that are required for the emulsion PCR and sequencing were added to the ends of the fragments by ligation. The resulting fragment library was sequenced on 1 picotiterplate (PTP) on the GS FLX using Roche/454 Titanium chemistry.

#### Assembly of the sequence reads to transcripts

Prior to assembly the sequence reads were screened for the SfiI linker used for concatenation, the linker sequences were clipped out of the reads and the clipped reads assembled to individual transcripts using the Roche/454 Newbler software (454 Life Sciences Corporation, software release 2.0.01.14) at the following parameter settings: seed step = 12; seed length = 16, minimum overlap length = 40, minimum overlap identity = 90%, alignment identity score = 2, alignment different score = -3. As a consequence, sequence reads were obtained using 454 (Roche) experimental procedures and materials, then sequence clusters were constructed using Newbler Assembler software (release 2.0.01.14) based on a sequence overlap threshold of 40 bases and an identity percentage of a least 90% within overlaps. Sequence clusters were aligned against reference databases for vectors [59], bacterial genomes [60], and mitochondria and chloroplast [61] as well as ribosomal [62] sequences. The 454 sequence data are publicly available at the National Center for Biotechnology Information [63] under accession numbers [JP206682] to [JP238633].

### Affymetrix array hybridization and analysis

#### Hybridization

The Affymetrix [64] wheat GeneChip^® ^oligonucleotide array, which have probes for 55,052 transcripts, was hybridized according to the following procedure. Total RNA (2 μg) from the five grain samples harvested in 2004 (109, 204, 247, 295, 501 DD) and 2006 (125, 186, 231, 292, 489 DD) were used to synthesize biotin-labeled cRNAs with the one-cycle cDNA synthesis kit (Affymetrix). SuperScript II reverse transcriptase and T7-oligo(dT) primers were used to synthesize single-stranded cDNA at 42°C for 1 hour, followed by synthesis of double-stranded cDNA using DNA ligase, DNA polymeraseI, and RNaseH for 2 h at 16°C. After cleaning of the double-stranded cDNA with the Sample Cleanup Module (Affymetrix), *in vitro *transcription was performed in the presence of biotin-labeled UTP using the GeneChip^® ^IVT labeling kit (Affymetrix). The labeled cDNA was purified with the Sample Cleanup Module (Affymetrix) and quantified with RiboGreen RNA quantification reagent (Turner Biosystems, [65]). Fragmentation of 15 μg of labeled cDNA was carried out for 35 minutes at 94°C, followed by hybridization for 16 hours at 45°C to Affymetrix wheat GeneChip^® ^oligonucleotide arrays. After hybridization, the arrays were washed with two different buffers (stringent: 6 SSPE, 0.01% Tween 20; and non-stringent: 100 mm MES, 0.1 M Na^+^, 0.01% Tween 20) and stained with a complex solution including Streptavidin R-Phycoerythrin conjugate (Molecular Probes, [66]) and anti-streptavidin biotinylated antibody (Vector Laboratories, [67]). The washing and staining steps were performed in a GeneChip^® ^Fluidics Station 450 (Affymetrix). The Affymetrix wheat GeneChip^® ^oligonucleotide arrays were finally scanned with the GeneChip^® ^Scanner 3000 7G piloted by GeneChip^® ^Operating software. All these steps were performed on an Affymetrix platform at INRA-URGV in Evry (France).

#### Statistical data analysis

The raw CEL files were imported in the Bioconductor software package in R for data analysis [68]. The data were normalized with the gcrma algorithm [69] available in the Bioconductor package. To determine differentially expressed genes, we performed a standard two-group *t*-test that assumes equal variance between groups. The variance of the gene expression per group is a homoscedastic variance, where genes displaying extremes of variance (too small or too large) were excluded. The raw *P*-values were adjusted by the Bonferroni method, which controls the familywise error rate [70]. A gene is declared as differentially expressed if the Bonferroni P-value is < 0.05. The raw data are available through the CATdb database (reference AFFY_seed_kinetic_Wheat) [71] and from the Gene Expression Omnibus [72] at the National Center for Biotechnology Information (NCBI), accession number GSE 16457.

### cDNA-SSCP primer design and profile analysis

#### Primer design

Affymetrix wheat GeneChip^® ^sequences were download from the Affymetrix online database [73] and used to design primers pairs. Wheat sequence exons structures were identified through rice/*Brachypodium*/sorghum/maize and wheat sequence alignments and provided to the Primer 3 package to select primer only on one exon using default parameters.

#### cDNA-SSCP protocol

The absence of contaminating genomic DNA in RNA samples was tested directly by PCR. cDNA was synthesized using Transcriptor First Strand cDNA Synthesis Kit (Roche) and diluted 50 times. PCR products were generated and analyzed with the SSCP protocol according to Quraishi *et al. *[26]. Briefly, cDNA-PCR fragments were produced in two steps. In a total volume of 15 μl, genomic DNA (30 ng) was first amplified with the following PCR mix: 10 mM Tris-HCL, 3.1 mM MgCl_2_, 50 mM KCl, 0.001% gelatine pH 8.3, 5% glycerol, 400 μM dNTP, 0.4 μM forward and reverse primers, 0.2 U Taq polymerase (Qiagen). This PCR product was diluted (1/10) and re-amplified with the same PCR mix, including 0.2 μM of each labeled primer (6-FAM and NED, Applied Biosystems [74]) in a final volume of 15 μl. The PCR product (2 μl) was then diluted (1/10) and pooled with 0.2 μl of 900 bp MegaBace ET900-R Size Standard (GE Healthcare, [75]), 0.2 μl of 0.3 N NaOH and 9 μl HI-Di Formamide (Applied Biosystems). Fragments were separated by capillary electrophoresis on an ABI3100 (Applied Biosystems) in 50 minutes with a 36 cm capillary. The running polymer consisted of 1× running buffer, 5% Genscan Polymer (Applied Biosystems), 10% glycerol. Samples were denatured for 2 minutes at 95°C and then 10 minutes in ice. The sample buffer consisted of 1× running buffer and 10% glycerol. After denaturation, the samples were injected at 2.5 kV over 50 seconds and separated at 18, 25, and 35°C and 15 kV. Data were analyzed using GeneMapper 3.7 software.

### Identification of homeologs, orthologs and paralogs in wheat genomes

The methodology used to reassess the synteny between wheat/rice/*Brachypodium*/sorghum/maize genomes as well as the identification of intra-chromosomal duplications in wheat is described in detail in Salse *et al. *[2,76], Bolot *et al. *[77], and Abrouk *et al. *[4]. Wheat (5,003 mapped unigene set), rice (41,046 genes), *Brachypodium *(25,504 genes), sorghum (34,008 genes) and maize (32,540 genes) genomes were aligned to identify orthologs and co-linear regions [2,3]. Three parameters were used to increase the stringency and significance of BLAST sequence alignment by parsing BLASTn results and rebuilding high scoring pairs (HSPs) or pairwise sequence alignments. The first parameter, aligned length (AL), corresponds to the sum of all HSP lengths. The second, cumulative identity percentage (CIP) corresponds to the cumulative percent of sequence identity obtained for all the HSPs (CIP = ∑ nb ID by HSP/AL) × 100). The third parameter is the cumulative alignment length percentage (CALP). It represents the sum of the HSP lengths (AL) for all the HSPs divided by the length of the query sequence (CALP = AL/Query length). The CIP and CALP parameters allow the identification of the best alignment, that is, the highest cumulative percentage of identity in the longest cumulative length, taking into account all HSPs obtained for any pairwise alignment. These parameters were applied to all the BLAST alignments that were performed in the present study. Based on the genome-wide synteny analysis, gene relationships between species are then referenced as COS (for conserved gene pairs), CNV (for tandem duplicated genes), PAV (for non-conserved genes).

## Abbreviations

AFLP: amplified fragment length polymorphism; bp: base pair; CALP: cumulative alignment length percentage; CNV: copy number variation; CDS: coding sequence; CIP: cumulative identity percentage; COS: conserved orthologous set; DD: degree day; EST: expressed sequence tag; GO: Gene Ontology; HSPs: high scoring pairs; MYA: million years ago; RFLP: restriction fragment length polymorphism; RNA-seq: RNA sequencing; SNP: single nucleotide polymorphism; SSCP: Single Strand Conformational Polymorphism; TF: transcription factor; WGD: whole genome duplication.

## Authors' contributions

Caroline Pont designed the experiment, performed the analysis and participated in manuscript preparation. Florent Murat performed the bioinfomatic analysis and participated in manuscript preparation. Carole Confolent performed the molecular biology experiments. Sandrine Balzergue performed the transcriptome (Affymetrix) analysis. Jérôme Salse designed the research program, managed the research group and wrote the article.

## Supplementary Material

Additional file 1 **Supplemenraty tables**. To support the use of the provided RNA-seq data, we provide eleven supplementary tables. Table S1: RNA-sequence quality and coverage features. Table S2: information on the 37,695 wheat sequence clusters. Table S3: the 17,881 homologs between wheat and *Brachypodium*/rice/sorghum/maize genomes. Table S4: the heterologous bread wheat expression map. Table S5: the SSCP analysis. Table S6: *Brachypodium *heat maps. Table S7: GO classification. Table S8: transcription factor data. Table S9: wheat Affymetrix experiment data. Table S10: the starch pathway analysis. Table S11: the structural/functional shuffling model. The provided supplementary tables provide access to the raw data (gene name, sequence, position, function, expression and statistical data) of the results detailed in the article. Click here for file
